# Hidden genomic MHC disparity between HLA-matched sibling pairs in hematopoietic stem cell transplantation

**DOI:** 10.1038/s41598-018-23682-y

**Published:** 2018-03-29

**Authors:** Satu Koskela, Jarmo Ritari, Kati Hyvärinen, Tony Kwan, Riitta Niittyvuopio, Maija Itälä-Remes, Tomi Pastinen, Jukka Partanen

**Affiliations:** 1 Research and Development, Finnish Red Cross Blood Service, Helsinki, Finland; 2 McGill University and Genome Quebec Innovation Centre, Montreal, Canada; 3 Helsinki University Hospital Comprehensive Cancer Center, Stem Cell Transplant Unit, Helsinki, Finland

## Abstract

Matching classical HLA alleles between donor and recipient is an important factor in avoiding adverse immunological effects in HSCT. Siblings with no differences in HLA alleles, either due to identical-by-state or identical-by-descent status, are considered to be optimal donors. We carried out a retrospective genomic sequence and SNP analysis of 336 fully HLA-A, -B, -DRB1 matched and 14 partially HLA-matched sibling HSCT pairs to determine the level of undetected mismatching within the MHC segment as well as to map their recombination sites. The genomic sequence of 34 genes locating in the MHC region revealed allelic mismatching at 1 to 8 additional genes in partially HLA-matched pairs. Also, fully matched pairs were found to have mismatching either at HLA-DPB1 or at non-HLA region within the MHC segment. Altogether, 3.9% of fully HLA-matched HSCT pairs had large genomic mismatching in the MHC segment. Recombination sites mapped to certain restricted locations. The number of mismatched nucleotides correlated with the risk of GvHD supporting the central role of full HLA matching in HSCT. High-density genome analysis revealed that fully HLA-matched siblings may not have identical MHC segments and even single allelic mismatching at any classical HLA gene often implies larger genomic differences along MHC.

## Introduction

Matching human leukocyte antigen (HLA) alleles between the donor and recipient of hematopoietic stem cell transplantation (HSCT) is crucial to reduce the risk of graft-versus-host disease (GvHD), a major life-threatening complication of HSCT. The donor search usually begins by HLA genotyping of immediate family members to find an HLA-identical sibling that is considered to be an optimal donor in the HSCT setting. Due to the low rate of intra-major histocompatibility complex (MHC) recombination, a patient and a sibling donor have a 25% chance to be HLA identical, i.e., they share the same two HLA haplotypes.

The segregation of all four parental HLA haplotypes in a family can often be defined on basis of HLA-A, -B, -DRB1 typing when either parents or sufficient number of siblings are available. As there is a strong linkage disequilibrium (LD) between HLA-B and -C genes and HLA-DRB1 and -DQB1 genes^1^, respectively, HLA-A,-B and -DRB1 matching usually implies also HLA-C and -DQB1 matching. However, due to the limited number of siblings and ever-older HSCT patients, all four haplotypes are not always distinguished in a family. Many HLA matched siblings can therefore be classified merely as HLA identical-by-state, rather than identical-by-descent. A chance for selecting only HLA matched but not HLA identical donor increases especially when similar haplotypes with the same HLA alleles in different combinations segregate in a family. To overcome these problems some histocompatibility laboratories now type larger than the minimal set of HLA genes.

In the absence of an HLA identical sibling donor, i.e. 6/6 HLA-matched donor, HLA-mismatched related donor with a single mismatch at HLA-A, -B or -DRB1 is an option and requires typing of at least one additional classical HLA gene, HLA-C or -DQB1 or -DPB1. However, these 5/6 HLA-matched sibling pairs may have allelic disparities at other classical HLA genes and/or elsewhere in the major histocompatibility complex (MHC). There is emerging evidence that the role of the MHC in GvHD susceptibility may be more complex than merely matching the alleles of the classical HLA genes. Some HLA-related or non-HLA variations within the MHC may be associated with GvHD risk^2–7^.

The major histocompatibility complex encompasses 4 Mbp of the short arm of chromosome 6p21.3. In addition to the classical HLA genes, non-classical HLA genes and a number of other genes, some with immunological functions, are found in this segment. A hallmark of the MHC is strong LD^8,9^. In many populations, a number of conserved extended or ancestral MHC haplotypes with a strong LD are present at a high frequency^10–13^. According to Baschal *et al*.^14^, conservation in a haplotype group varies greatly, but one-quarter of haplotype groups have greater than 50% conservation from HLA-DRB1 to HLA-A segment. For example, the common European HLA-A1-B8-DR3 haplotype is highly congruent, with 92% of chromosomes having 99.8% allelic identity.

Despite of the strong LD, a few recombination hot spots are located within the MHC region^15,16^. For example, a dense cluster of recombination hot spots maps to a segment between HLA-DQB1 and HLA-DPB1 genes in the MHC class II, resulting in a high recombination rate between these genes. In contrast, the segment between HLA-DRB1 and HLA-DQB1 genes, which has a similar physical length, has a very low recombination rate^13,17,18^. Active recombination sites have also been observed telomeric to HLA-A gene in the MHC class I and between HLA-B and HLA-DRB1 genes in the MHC class III region^19,20^. The locations of recombination hot spots have been found to be the same across populations, although some appear to be haplotype or population specific. These recombination sites lead to segmented blocks in the MHC.

Very few studies have used systematic genomic approaches to estimate the level of the MHC identity in HLA-matched sibling HSCTs^21^. The present genomic study can help us to assess whether current HLA matching protocols, in particular the set of HLA genes genotyped, are sufficient and how much there is hidden mismatching in the non-HLA MHC region. Furthermore, we can estimate the level of intra-MHC recombination and their boundaries. To address the question of genomic MHC identity in the HLA and non-HLA gene regions, we performed a retrospective analysis of 5137 single nucleotide polymorphisms (SNPs) located in the MHC segment in 261 HLA-matched sibling allogeneic HSCTs. A more detailed comparison utilizing the full genomic sequence of the MHC was performed in another set of 89 potential sibling HSCT pairs. The high-density SNP data together with the full MHC sequence analysis created an opportunity to uncover hidden mismatches within the MHC segment in apparently HLA-matched sibling HSCTs.

## Results

### Study cohort 1

Altogether, 261 pairs with a patient and his/her HLA typed sibling were included in the study. No parents were available for clinical HLA typing. Therefore, in most cases only HLA identical-by-state status, rather than identical-by-descent status, could be assigned for the HSCT patient-donor pairs.

### HLA match status and imputation

Based on the clinical HLA typing of five classical HLA genes a total of 255 of 261 pairs were 6/6 HLA-matched (HLA-A, -B, -DRB1). Six pairs were known to be 5/6 HLA-matched prior to the present study. Antigen mismatching occurred at HLA-A in three pairs, at HLA-B in two pairs and at HLA-DRB1 in one pair. One of the two pairs with mismatching at HLA-B had mismatching also at HLA-C and the pair with an antigen mismatch at HLA-DRB1 was mismatched also at HLA-DQB1 (Table 1).

**Table 1:** HLA-mismatched haematopoietic stem cell transplantation (HSCT) pairs of study cohort 1 (N = 261 sibling pairs) analysed by Immunochip single nucleotide polymorphism (SNP) array.

Pair id	Clinical HLA typing		Imputation of HLA-A,-B,-C,-DRB1,-DQA1,-DQB1 and -DPB1 alleles	Genotyping of 5137 SNPs within 4 Mbp region of MHC	Total match	GvHD grading	
	HLA-A, -B, -DRB1 match	Mismatched HLA allele	Mismatched HLA allele	SNP mismatching in MHC		Acute	Chronic
2329	5/6	DRB1, DQB1	DRB1, DQB1, DQA1, DPB1	C4 to DPB1	11/14	na	na
3205	5/6	A	A, DPB1	A, DPB1	12/14	2	limited
4426	5/6	A	A, DPB1	A, DPB1	12/14	0	extensive
4658	5/6	A	A	A	13/14	2	no
4754	5/6	B	B	C4 to HLA-E, telomeric to A	12/14	0	no
5366	5/6	B,C	B,C, DPB1	B to C, DPB1	11/14	na	na
1812	6/6	—	DPB1	DPB1	13/14	0	na
3450	6/6	—	DPB1	DPB1	13/14	0	no
4152	6/6	—	DPB1	DPB1	13/14	0	no
5236	6/6	—	DPB1	DPB1	13/14	0	no
2934	6/6	—	—	centromeric to DPB1	14/14	1	extensive
3446	6/6	—	—	centromeric to DPB1	14/14	1	limited
4706	6/6	—	—	centromeric to DPB1	14/14	0	limited
3803	6/6	—	—	telomeric to A	14/14	0	extensive
4205	6/6	—	—	non-HLA regions	14/14	3	limited

Six pairs were known to have one mismatched allele at HLA-A, -B or -DRB1 gene based on clinical HLA typing prior to HSCT. Four additional pairs were revealed to be mismatched by HLA allele imputation based on SNP data. Disparity outside the classical HLA genes in five pairs was uncovered by comparison of 5137 SNPs in the major histocompatibility complex region (MHC). HLA = human leukocyte antigen; SNP = single nucleotide polymorphism; MHC = major histocompatibility complex; C4 = complement component 4; GvHD = graft versus host disease.

To estimate the level of identity-by-state in seven classical HLA genes HLA-A, -B, -C, -DRB1, -DQA1, -DQB1 and -DPB1 in each 261 HSCT pair, we used an Immunochip array to screen SNP mismatches within the MHC. We explored the actual HLA match status of the six 5/6 HLA-matched HSCT pairs by imputing HLA alleles to the resolution level of unique amino acid chains (four-digit resolution).

Imputed four-digit HLA alleles were in 100% concordance with two-digit clinical HLA types. The number of imputed alleles varied between 48 and 1646 depending on the specific locus. Absolute posterior probability Q2 for HLA-A, -B, -C, -DRB1, -DQA1, -DQB1 and -DPB1 varied between 0.54–1.00, 0.19–1.00, 0.17–1.00, 0.19–1.00, 0.35–1.00, 0.46–1.00 and 0.12–1.00, respectively. The differences of Q2 values within HLA-mismatched pairs were relatively low: 0.00–0.01 for HLA-A, 0.00–0.28 for HLA-B, 0.17 for HLA-C, 0.07 for HLA-DRB1, 0.44 for HLA-DQA1, 0.01 for HLA-DQB1 and 0.01–0.46 for HLA-DPB1. Metrics of the imputation and imputed HLA alleles for each pair are described in more detail in Supplementary Table S1.

Imputation revealed additional mismatching at those classical HLA genes not typed prior to transplantation: pair 2329, which was known to have allelic mismatches at HLA-DRB1 and -DQB1, was also mismatched at HLA-DQA1, and four pairs with allelic mismatching at HLA-A or -B were also mismatched at HLA-DPB1 (pairs 2329, 3205, 4426 and 5366 in Table 1 and Supplementary Table S2). Furthermore, mismatching at HLA-DPB1 was revealed in four out of 255 fully (6/6) HLA-matched pairs (pairs 1812, 3450, 4152 and 5236 in Table 1). Subsequent HLA typing by the reference technique SSO confirmed allelic mismatching at HLA-DPB1 in all the pairs from which DNA was available, that is, five out of eight DPB1-mismatched pairs. In total, 10 of the 261 (3.8%) HSCTs in study cohort 1 were performed between pairs with at least one allelic mismatch in the classical HLA genes.

### MHC segment identity

**Figure 1:**
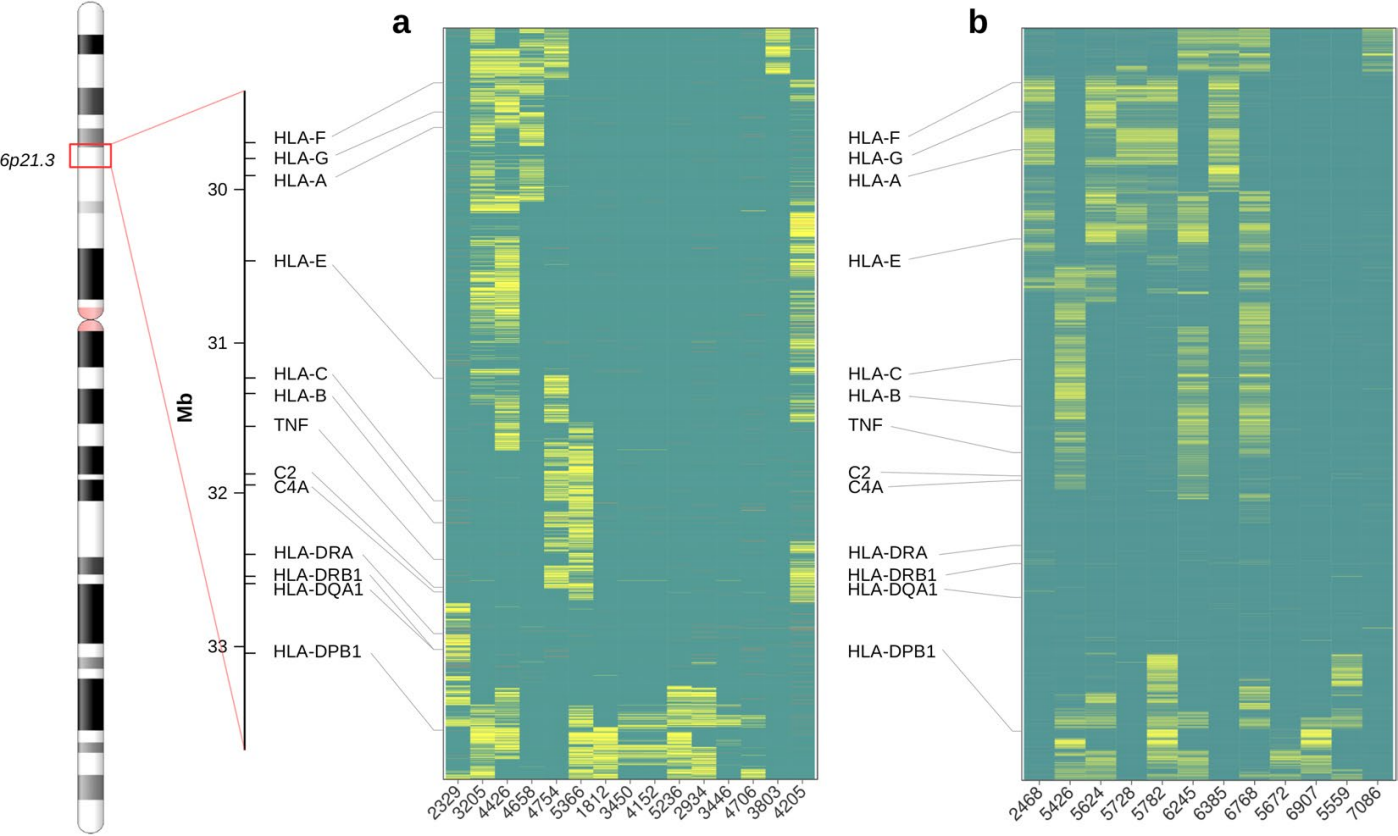
Genotype comparison map showing haematopoietic stem cell transplantation (HSCT) donor-recipient pairs with large genomic fragments of mismatched single nucleotide polymorphisms (SNPs) in the major histocompatibility complex (MHC) segment. The mismatched HSCT pairs are shown in discovery order on the X-axis. Green depicts matched SNPs, yellow depicts mismatched and red indicates missing results or poor quality SNPs. Physical map of the MHC region with selected marker genes is shown on the left. The vertical axes in the similarity plot show the positions of SNP having a mismatch in at least one pair. (**a**) Identical-by-state status of 5137 SNPs covering the entire MHC gene complex by an Immunochip array in 15 HSCT sibling pairs (study cohort 1). The Y-axis shows a segment of chromosome 6p21 encompassing positions 29,002,062 bp (telomeric to HLA-F) to 33,496,714 bp (centromeric to HLA-DPB1). (**b**) Identical-by-state status of 12 HSCT pairs sequenced for the 4.5 Mbp MHC region (study cohort 2). The Y-axis shows a segment of chromosome 6p21 encompassing positions 29,000,825 bp (telomeric to HLA-F) to 33,477,140 bp (centromeric to HLA-DPB1).

We next scrutinized the level of overall SNP matching within the entire MHC region in each of the 261 HSCT pairs based on the matching of the 5137 SNPs that had been mapped to that segment. Figure 1a shows the matched (dark green) and mismatched (yellow) SNPs in HSCT pairs with large mismatching fragments. The mismatched fragments covered those HLA genes that were known to have allelic mismatches by clinical HLA typing or HLA imputation. Thus, the 10 HSCT pairs with identified mismatching at the classical HLA had mismatched SNP clusters especially at the particular gene with an allele mismatch. The mismatched regions, however, covered larger areas than only the exact HLA gene with allelic mismatching. For example, pair 2329, which was originally known to be mismatched at HLA-DRB1 and -DQB1 alleles, showed SNP mismatches encompassing the entire segment from complement C4 gene to the telomeric side of HLA-DPB1. In two HLA-B allele mismatched pairs (pairs 4754 and 5366 in Fig. 1a), SNP mismatching extended close to complement C4 gene at the centromeric side of HLA-B and close to HLA-E at the telomeric side. Pair 4754 additionally had SNP mismatching telomeric to HLA-A gene. In all three pairs with mismatched HLA-A alleles (pairs 3205, 4426, 4658) SNP mismatched areas were large extending far from HLA-A gene in both directions. Mismatching at HLA-DPB1 reached far to the centromeric side of the gene in six mismatched pairs (3205, 5366, 1812, 2934, 5236, 4706). The number of mismatched SNPs and putative boundaries of the recombination segments are listed in Supplementary Table S3.

In addition to the 10 mismatched pairs described above, four other HSCT pairs had clusters of mismatched SNPs within the MHC. In other words, they were found to be not identical-by-state. These SNP mismatches were located telomeric to HLA-A or centromeric to HLA-DPB1 (pairs 2934, 3446, 4706, 3803 in Fig. 1a) covering the genomic area where non-classical HLA genes or HLA pseudogenes reside: HLA-G and -F genes at the telomeric end of the MHC and HLA-DPB2 at the other end. Surprisingly, pair 4205, which had no HLA allele disparities in the seven classical HLA genes (14/14 match), was found to have clusters of mismatched SNPs in the MHC. Large regions telomeric to HLA-A, between HLA-A and HLA-C and between HLA-B and DRB1, encompassing the entire TNF-C4 fragment in the MHC class III, were covered with SNP mismatches. Taken those mismatches in the MHC region into account that were not known before the transplantation, altogether 3.5% (9/255) of the HSCT pairs in study cohort 1 were mismatched.

The remaining 246 fully 6/6 HLA-matched HSCT sibling pairs had no known HLA allelic mismatches or SNP mismatches, barring some apparently sporadic single SNP differences (Supplementary Fig. S1).

### Study cohort 2

Study cohort 2 consisted of 89 HLA-matched sibling HSCT pairs, of which five were known to have a single allelic HLA-A mismatch and three to have HLA-B and -C mismatch. None of them had mismatching at HLA-DRB1 (Table 2).

**Table 2:** HLA-mismatched haematopoietic stem cell transplantation (HSCT) pairs in study cohort 2 (N = 89 sibling pairs) analysed by genomic sequencing of the major histocompatibility complex (MHC) region.

Pair id	Clinical HLA typing		NGS-HLA allele assignment of HLA-A,-B,-C,-DRB1,-DQA1, -DQB1 and -DPB1 genes	Determination of genomic 4.5 Mb sequence of the MHC region	Total match	GvHD grading	
	HLA-A,-B,-DRB1 match	Mismatched HLA allele	Mismatched HLA allele	HLA and non-HLA gene sequence mismatching		Acute	Chronic
2468	5/6	A	A	A, F, H, K	30/34	1	extensive
5426	5/6	B,C	B, C, DPB1	B, C, MICA, TAP1, DPB1, DPB2	28/34	0	no
5624	5/6	A	A	A, H, TAP2, TAP1, DMA	29/34	na	na
5728	5/6	A	A	A, F, H, K, L, E	28/34	0	limited
5782	5/6	A	A, DPB1	A, F, H, K, DOB, TAP2, DPA1, DPB1	26/34	na	na
6245	5/6	B,C	B, C	B, C, L, MICA, MICB	29/34	na	na
6385	5/6	A	A	A, G, H, K, TAP2	29/34	na	na
6768	5/6	B,C	B, C	B, C, E, MICA, DOB, TAP2, TAP1	27/34	1	extensive
5672	6/6	—	DPB1	DPB1, DOB	32/34	na	na
6907	6/6	—	DPB1	DPB1, DPA1	32/34	na	na
5559	6/6	—	—	centromeric and telomeric to DPB2	34/34	na	na
7086	6/6	—	—	telomeric to F	34/34	0	limited

Eight pairs were known to have one allelic mismatch at HLA-A, -B or -DRB1 gene based on clinical HLA typing prior to HSCT. Allelic mismatching at HLA-DPB1 was revealed in two pairs by next generation sequencing allele assignment. Two more pairs were uncovered to be mismatched by genomic sequence comparison of the entire MHC segment. Only those genes with mismatching at the amino acid level are shown. + . HLA = human leukocyte antigen; MHC = major histocompatibility complex; MICA = MHC class I chain-related gene A; MICB = MHC class I chain-related gene B; TAP1 = transporter 1, ATP binding cassette subfamily B member; TAP2 = transporter 2, ATP binding cassette subfamily B member; GvHD = graft versus host disease.

### HLA match status

The full genomic MHC segment (4.5 Mbp) was sequenced from all 89 HSCT pairs. Alleles of the classical HLA genes HLA-A, -C, -B, -DRB1, -DQA1, -DQB1, and -DPB1 were assigned based on the genomic sequence using Omixon Explore 1.2.0 at four-digit and six-digit resolution levels (missense and silent mutations). There was 100% concordance to the clinical HLA-A, -C, -B, -DRB1, -DQB1 typing. As in cohort 1, additional disparities at HLA-DPB1 were found in two 5/6 HLA-matched HSCT pairs (pairs 5426, 5782 in Table 2). No allelic disparities were found at HLA-DRB1 or -DQA1.

**Figure 2:**
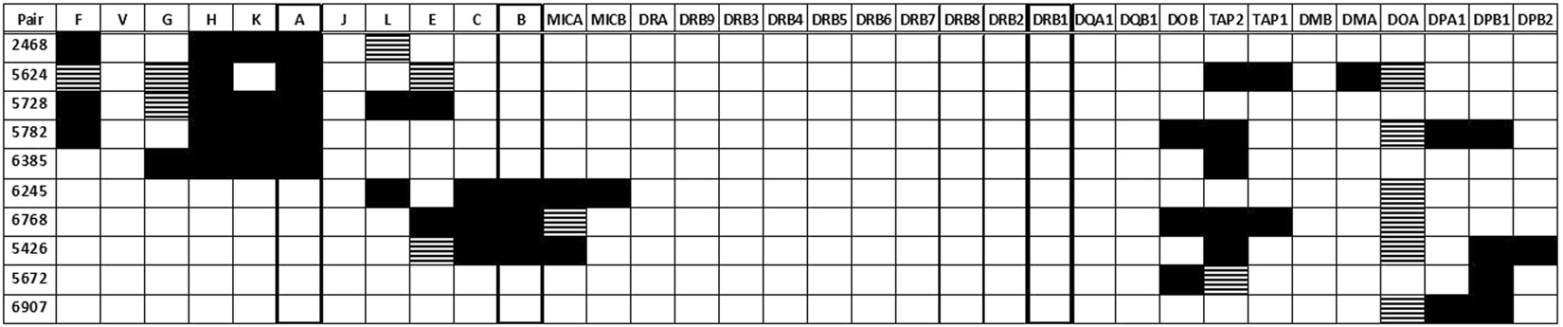
Allelic mismatching in cohort 2 of 34 genes located in the major histocompatibility complex region. The genes are listed from the telomeric end (HLA-F) to the centromeric end (HLA-DPB2). The classical HLA-A, -B, -C, -DRB1 and -DQB1 genes are highlighted. The eight uppermost haematopoietic stem cell transplantation pairs are 5/6 HLA-matched, and pairs 5672 and 6907 are 6/6 HLA-matched siblings. Alleles of the 34 genes are assigned based on exon sequence analysis at six-digit resolution level. Allelic mismatching that results in amino acid change is depicted in grey. Mismatching that remains at the DNA level (silent mutations) is marked with stripes.

Furthermore, the alleles of 27 additional genes in the MHC region were assigned in pairs with known HLA mismatching. These genes included non-classical HLA genes, HLA pseudogenes and non-HLA genes: HLA-F, -V, -G, -H, -K, -J, -L, -E, -DRA, -DRB2-9, -DOB, -DMB, -DMA, -DOA, -DPA1, -DPB2, MICA, MICB, TAP1 and TAP2. In the 5/6 HLA-matched pairs, the number of additional genes with allelic mismatches leading to a change at the amino acid level varied between three and seven (Table 2). Thus, the minimum number of genes with allelic disparities at four-digit resolution was four (pair 2468) and the maximum number was as high as eight (pair 5782). The matching status including all 34 MHC genes varied between 26/34 and 30/34. As expected, the number of mismatching was higher at the DNA level (six-digits), varying between five and nine (Fig. 2). All five pairs with mismatching at HLA-A had also mismatching at HLA-F, -G, -H, and/or -K located telomeric to HLA-A gene. Likewise, all pairs with HLA-B mismatching also had MICA mismatching and one pair also had MICB mismatching. In addition, some extra mismatching in these pairs occurred at other genes of the MHC class I. Interestingly, many pairs with allelic mismatching in the MHC class I also had allelic disparities in MHC class II genes centromeric to HLA-DQB1 gene. In this study cohort, no allelic HLA-DQA1 or -DQB1 mismatching was found, most probably due to the tight LD of the matched HLA-DRB1 alleles.

Consistent with the results of study cohort 1, not all of the 6/6 HLA-matched pairs were fully matched for the entire MHC. Pair 5672 had allelic mismatching at HLA-DPB1, -DOB and TAP2, and pair 6907 had mismatching at HLA-DPB1 together with HLA-DPA1 and -DOA (Table 2 and Fig. 2). In this cohort 8.9% (10/89) of the HSCT pairs were mismatched at least for one classical HLA allele.

### MHC segment identity

The extent of the mismatched fragments in the 89 HSCT pairs was examined further at the genomic sequence level (Fig. 1b). Again, the mismatched genomic regions covered larger segments around the particular gene with observed allelic HLA mismatching. Pairs 5426, 6245, and 6768 mismatched both at HLA-B and HLA-C showed mismatching at genomic segment starting from the telomeric side of HLA-C gene and ending centromeric to complement C4 gene in the MHC class III. The mismatched region in the pairs with allelic HLA-A disparity (pairs 2468, 5624, 5728, 5782, 6385) encompassed relatively long segments flanking the entire HLA-A gene. Two samples, 5559 and 7086, showed MHC fragment mismatching despite of having no mismatching at any of the 34 genes (Table 2 and Fig. 1b). The observed mismatching located centromeric to HLA-DPB2 gene in pair 5559 and telomeric to HLA-F gene in pair 7086. It is of note that approximately the same segments were also mismatched in some of the 5/6 HLA-matched pairs as well (5782, 6245, 6768, 7086). Altogether 4.9% (4/81) of the HLA matched HSCT pairs in study cohort 2 were found to have unexpected disparities in the MHC region. The remaining 77 fully HLA-matched HSCT sibling pairs were identical-by-state along the entire 4.5 Mbp MHC region, barring some sporadic single mismatched nucleotides (Supplementary Fig. S1).

Taking the two cohorts together, 7.7% (27/350) of the putative HSCT pairs were found to have mismatches in the MHC region of which 3.9% (13/336) were observed in fully HLA-A, -B, -C, -DRB1, -DQB1 matched pairs.

### Recombination sites

The positions of the mismatched fragments were examined in both study cohorts (Supplementary Table S3). The boundaries of the mismatched segments were focused on restricted genomic regions and appeared to be non-random. The putative recombination sites in the MHC class I region clustered around position ~29.6 Mbp just telomeric to HLA-F gene and in the 1.0 Mbp segment around positions 30.0 Mbp and 31.0 Mbp, close to HLA-C gene, in the HLA-A mismatched pairs. The recombination sites between the MHC class I and class II clustered around position ~32.1 Mbp locating centromeric to complement C4 gene. In the MHC class II region, the recombination sites between HLA-DQB1 and -DPB1 genes clustered within a ~200-Kbp segment between positions 32.8 Mbp and 33.0 Mbp. All pairs with allelic mismatching at HLA-DPB1showed recombination in a surprisingly narrow segment of ~400 Kbp between positions 32.7 Mbp and 33.1 Mbp.

### Effect of mismatching on risk of graft-versus-host disease

**Figure 3:**
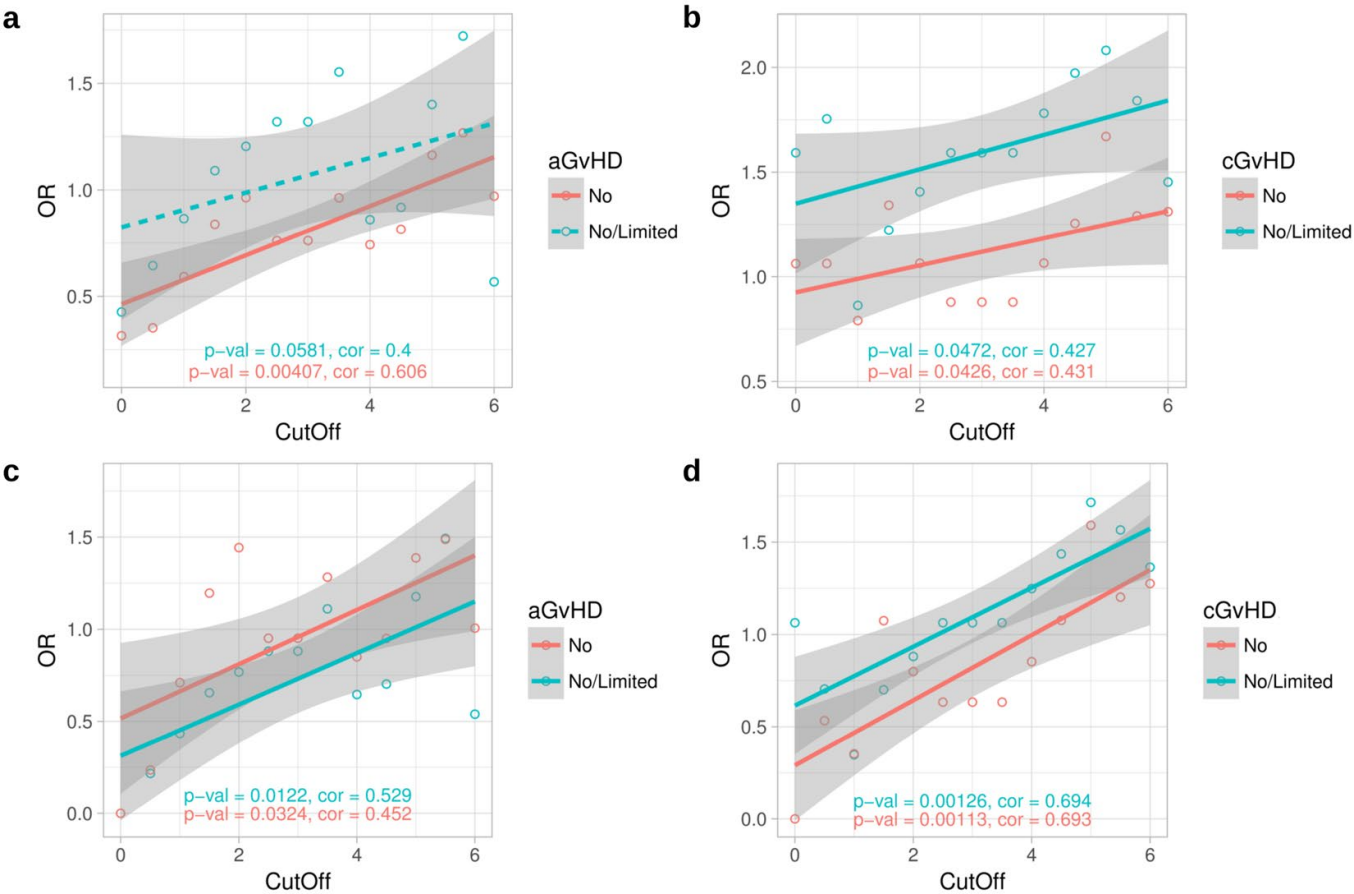
Trend toward higher risk for graft-versus-host disease (GvHD) along increasing number of nucleotide mismatches between haematopoietic stem cell transplantation (HSCT) donor-recipient pairs in study cohort 1. The donor-recipient pairs are divided into low and high mismatch groups according to the total number of MHC region genotype differences between each pair. Similarly, each pair is also assigned into either aGvHD positive or negative group according to the recipient’s clinical GvHD gradus. The mismatch and GvHD categorized data are then arranged into a contingency table to calculate the odds ratio (OR). The mismatch threshold value, defined as the natural logarithm of the number of total SNP genotype differences per each HSCT sibling pair, is varied from 0 to 6 and the corresponding odds ratio is calculated for each threshold value. Thus, each data point represents an odds ratio at a particular threshold value. Moreover, two alternate definitions for the GvHD negative status are used: grade 0 (no) or grades 0–2 (no/limited) in acute GvHD, and no GvHD or no/limited in the chronic GvHD. The plots show the odds ratios (y-axis) against the varying mismatch threshold values (x-axis). (**a**) Acute (n = 91) and (**b**) chronic (n = 62) GvHD for cohort 1, and (**c**) acute (n = 85) and (**d**) chronic (n = 56) GvHD for fully HLA A-B-DRB1 matched cohort 1 pairs. Pairs with zero MHC mismatches are omitted. Linear regression lines are shown for both GvHD negative groups by their corresponding colours. Correlation is calculated by Kendall’s rank correlation. aGvHD, acute graft-versus-host disease; cGvHD chronic graft-versus-host disease; OR, odds ratio.

The number of subjects with clinical information was 255 for acute GvHD (no = 172, grade 1 or 2 = 58; grade 3 or 4 = 25) and 202 for chronic GvHD (no = 98; limited = 52; extensive = 52). Of these, MHC sequence mismatching occurred in 91 aGvHD cases and 62 cGvHD cases, and out of mismatched aGvHD and cGvHD cases 85 and 56 were fully HLA-A, -B, -DRB1 matched, respectively. We found a weak but consistent trend between the number of mismatched nucleotides within the MHC segment and the risk of both acute and chronic GvHD as estimated by odds ratio vs. mismatch threshold (Fig. 3a,b). The trend remained significant after excluding 5/6 HLA-matched pairs (Fig. 3c,d).

## Discussion

In the present study, we used SNP genotyping and genomic sequencing to investigate the matching of the entire MHC region in 350 fully or partially HLA-A, -B and -DRB1 matched sibling donor-recipient HSCT pairs. The basic questions addressed in the study were: how frequently do HLA-A, -B, -DRB1 matched pairs have mismatching in the MHC region, how large genomic fragments do them cover and does the level of mismatching correlate with the occurrence of graft-versus-host disease? Altogether, 7.7% of all HSCT pairs and 3.9% of those pairs without a prior mismatch at HLA-A, -B or -DRB1 were found to have genomic differences in the MHC segment. Hence, hidden mismatching at HLA and non-HLA regions in the MHC were uncovered not only in 5/6 HLA-matched pairs but also in 6/6 HLA-matched pairs. The mismatched genomic fragments were larger than just a single HLA gene with allelic mismatch, sometimes covering many flanking genes. It is of note that our material was retrospective and HLA typing and matching were done as recommended during the years 1993–2011. Currently, the technological advance, in particular, has led to typing of a wider HLA profile in some laboratories. Importantly, despite of the limited sample size in this study, the number of mismatched SNPs showed a positive association with the risks of acute and chronic GvHD even after excluding the cases with known prior mismatching at HLA-A, -B, or DRB1. This result supports the primary role of matching the HLA segment in HSCT.

We determined the allelic variation in 34 genes located in the MHC and found that some pairs were only matched for 26 of these genes. Allelic mismatching at HLA-A usually resulted in mismatching at the non-classical HLA-F and -G as well as at pseudogenes HLA-H and -K. Likewise, mismatching at HLA-B encompassed mismatching at HLA-C and MICA and in one sample also at MICB. Allelic mismatching at HLA-DPB1 gene were relatively common, independently or together with mismatching elsewhere and, interestingly, usually also included TAP1 or TAP2 genes. In addition, we found a few cases with isolated allelic mismatching at HLA-DOB, TAP1, TAP2, HLA-DMA and HLA-DOA genes, without detectable mismatching at other MHC class II genes, indicating either highly-similar but different haplotypes or intra-MHC recombination. It seems that the functional consequences of a single allelic HLA mismatch can be wider than assumed. If all haplotypes in a family are not known, genotyping of the seven most important classical HLA genes in every sibling pair setting should be performed to ensure the HLA identity between a transplant pair. This action would only ensure the HLA identity but not the haplotype identity, as mismatches were also observed in large areas of the intervening sequence. To ascertain the haplotype identity a high-density SNP panel covering the entire MHC would be needed.

There is evidence that variations in HLA-DPB1, -G, -E and MICA genes are associated with immunological diseases, including also reports on graft-versus-host diseases^5,22–26^ and graft rejection^27^. The role of allelic mismatching at HLA-DPB1 in HSCT appears to be complex as it is related to expression level^23^ and so called permissive and non-permissive mismatch groups^22,27,28^. It is also known that even a single allelic mismatch at HLA can induce an alloimmune response^29,30^, including graft-versus-leukemia effect, a favourable phenomenon that reduces the risk of relapse^31,32^. Hence, our finding that the number of mismatched nucleotides correlates with the GvHD risk fits well to these findings.

The MHC region is known to have relatively conserved genomic blocks of a few to hundreds of thousands of kilobases in length. These blocks are flanked by recombination sites or hot spots^33,34^ resulting in the sharing of these segments by many unrelated individuals or different haplotypes. The borders of the mismatched segments observed in 27 pairs of this study were in agreement with the published recombination sites within the MHC class I, II and III regions^15,16^. Sometimes MHC blocks may span several megabases of DNA, covering almost the entire MHC fragment and are referred to as ancestral or conserved extended MHC haplotypes (AH or CEH)^10–13^. These long and fixed haplotypes are remarkably conserved, having a high level of allele identity across the MHC. For example, the most frequent North European HLA haplotype, 8.1 AH (HLA-A1-B8-DR3), is 92–98% congruent, but some polymorphisms are found telomeric to HLA-A and centromeric to HLA-DQB1^35^. The level of congruence can vary greatly depending on the haplotype group, especially if they are not AHs^14^. It is therefore possible that there are many different copies of the same haplotype in a family and that fully HLA-matched siblings are not haplotype identical^36^. This may explain SNP mismatching in the non-HLA MHC regions of the seven 6/6 HLA-matched pairs although meiotic recombination cannot be excluded.

As no parents or children were available for HLA typing due to the clinical practice in Finland, the sibling pairs could be only classified identical-by-state but not by-descent. It is therefore not possible to know whether mismatching between 5/6 HLA-matched siblings was due to meiotic recombination or because the siblings had inherited different but very similar haplotypes with only one allelic difference at HLA. For example, two similar haplotypes that are very frequent in the Finnish population, A*02:01-C*07:02-B*07:2-DRB1*15:01-DQB1*06:02 and A*03:01-C*07:02-B*07:2-DRB1*15:01-DQB1*06:02, can readily occur within a single family. Alternatively, parental chromosomes may by chance carry the same HLA alleles, but in completely different haplotypes, i.e., siblings have inherited different chromosomes from their parents. This may be the case in pair 4205, which was identical at the classical HLA-A, -B, and -DRB1 genes, but large segments with mismatched SNPs were observed in the sequence between them. The frequencies of the putative haplotypes that can be formed from the pair’s HLA combinations (HLA-A*02:01, 11:01; C*03:03, 03:03; B*15:01, 55:01; DRB1*13:01, 14:01; DQB1*05:03, 06:03) vary between 0.014 and 0.0003 in the Finnish population according to our unpublished results, which ranks 10^th^–412^th^ in haplotype frequency.

To our knowledge, this type of study in which MHC sequences between sibling pairs are scrutinised in detail has not been performed before. The discovery of hidden mismatches within the MHC that can encompass many genes, even in fully HLA-matched HSCT pairs, could encourage HLA laboratories to screen the MHC region more thoroughly with new DNA techniques in particular as there is a trend towards higher risk of GvHD along the number of mismatched nucleotides. The present study also provides useful information on the extent of MHC variation, which is important as haploidentical HSCTs are now becoming more into practice.

## Material and Methods

### Study cohorts and DNA extraction

Two different study cohorts were examined. Study cohort 1 was composed of 261 HSCT sibling pairs and cohort 2 of 89 possible HSCT sibling pairs. The actual transplantations of the cohorts were done between the years 1993 and 2011. Genomic DNA from the white blood cell fraction of whole blood was extracted with QiaAmp Blood minikit columns (Qiagen GmbH, Germany). This study was carried out in accordance with the recommendations of the Ethical Committee of Helsinki University Hospital with written informed consent from living subjects. The authority operating under the Ministry of Social Affairs and Health, Valvira, approved the study for deceased subjects. Demographic details and clinical outcomes including GvHD grading of HSCT pairs of cohort 1 are described in Supplementary Table S4 and in our previous studies^37,38^.

### Clinical HLA typing

Clinical HLA typing was performed at the HLA Laboratory of the Finnish Red Cross Blood Service using procedures accredited by the European Federation for Immunogenetics (EFI). All patients and donors of cohort 1, whose HSCT were done between the years 1993–2006, were typed for HLA-A, -B and -DRB1 either by the serological method (Lymphotype HLA-AB and Lymphotype HLA-DR, Bio-Rad Medical Diagnostics, Dreieich, Germany) or by PCR-based typing methods at two-digit resolution level (a LIPA reverse dot blot kit, Innogenetics Group, Gent, Belgium or Pel Freez HLA-SSP kits, Dynal Biotech LLC, Oslo, Norway). Depending on a given time period HLA-C and/or -DQB1 genes were also genotyped at four-digit resolution level in the pairs with antigen mismatching at any of HLA-A, -B or -DRB1 (5/6 HLA-matched pairs) conforming to the requirements given by EFI effective at the time.

Study cohort 2, whose HSCT were done between the years 2006–2011, was HLA-A, -B, -C, -DQB1 and -DRB1 genotyped at two-digit resolution level by rSSO-Luminex technology (Labtype, One Lambda, Inc., CA, USA) and PCR-SSP (Micro SSP™ Generic HLA Class I/II DNA Typing Trays, One Lambda, Inc.; Olerup SSP® genotyping, Olerup SSP AB, Stockholm, Sweden). The 5/6 HLA-matched pairs were retyped by sequence-based typing at four-digit resolution level (AlleleSEQR PCR/Sequencing kits, Atria Genetics, Hayward, CA, USA) using ABI 3130xl genetic analyzer (Applied Biosystems, Thermo Fisher Scientific, MA, USA). The results were analysed with Assign 3.5+ software (Conexio Genomics Pty Ltd, Fremantle, Australia.

Depending on the recommendations by EFI at any given time period, the clinical HLA typing did not result in the homogeneous set of HLA genes typed and/or the assignation of the alleles at the same resolution. To simplify the analyses, antigenic matching at HLA-A, -B, -C, -DRB1 and -DQB1 was set as the starting point.

### Immunochip array

Study cohort 1 was genotyped at the Institute for Molecular Medicine Finland, University of Helsinki, by using Immunochip array (Illumina, Inc., CA, USA). The Immunochip included 8215 SNPs within the DNA segment from the telomeric side of HLA-F gene to the centromeric side of HLA-DPB2 gene at positions 29–33.5 Mbp (Genome Reference Consortium human [GRCh]37/hg19). The initial quality control identified samples with discordant sex information, duplicate samples, call rate <97%, and heterozygosity excess <−0.3 (not X chromosome) or >0.2, and >0.1 for the X chromosome. The data were quality-filtered according to Anderson *et al*.^39^. After quality controls, 5137 SNPs were included in the study.

The alleles of the classical HLA-A, -B, -C, -DRB1, -DQA1, -DQB1 and -DPB1 genes were imputed at four-digit resolution level using the software HLA*IMP:02^40^ (The Oxford HLA Imputation Framework, UK). Missing data threshold was set to 0.20. SNPs were aligned and genotypes phased against HapMap (CEU) reference panel (hapmap3_r27_b36_fwd.consensus.qc.poly.chr6_ceu.). The absolute posterior probability scores Q2 for each imputed genotype were produced by using threshold T = 0.00.

### Genomic sequencing

Genomic sequencing of the full MHC region in samples of cohort 2 was performed at the McGill Genome Centre, McGill University, Montreal, Canada, using Roche SeqCap EZ Human MHC Design capture, which captures approximately 95% of the MHC region^41^. Sequencing was performed with an Illumina HiSeq. 2000, yielding 100 bp paired-end reads and a median on-target coverage of 27.5 × per sample. The generated reads were aligned to the GRCH37/hg19 reference genome. Base quality score recalibration and SNP and INDEL discovery were performed using GATK v.3.6-0 VariantRecalibrator and ApplyRecalibration tools with the default settings. The used *ts_filter_level* setting was 99.0^42–44^. The data were filtered using the hard cutoffs of the total depth of coverage per sample >7 and genomic quality >19^45^.

For HLA typing, the fastq read data were quality checked using FastQC^46^, and adapters were trimmed using Cutadapt^47^. The Omixon Explore program version 1.2.0 (Omixon, Budapest, Hungary) was used for allele assignment of 30 genes at six-digit resolution level comprising both classical HLA genes, non-classical HLA genes and pseudogenes in the MHC region using the IMGT/HLA database’s HLA nomenclature release 3.25.0: HLA-F, HLA-V, HLA-G, HLA-H, HLA-K, HLA-A, HLA-J, HLA-L, HLA-E, HLA-C, HLA-B, HLA-DRA, HLA-DRB1-9, HLA-DQA1, HLA-DQB1, HLA-DOB, HLA-DMB, HLA-DMA, HLA-DOA, HLA-DPA1, HLA-DPB1, HLA-DPB2. In addition, the alleles of the two MHC class I chain-related genes MICA and MICB as well as two ATP binding cassette transporter genes, TAP1 and TAP2, were assigned.

### MHC mismatch analysis

The genotypes of each HSCT sibling donor-recipient pair were compared position-by-position over the segment 29–33.5 Mbp of chromosome 6p21.3 by classifying each position into to one of three categories: the diploid genotype in a given position between pairs was identical, not identical or missing. Genotype positions with missing calls of over 5% were removed before the comparison. For study cohort 1, which was genotyped with Immunochip, the data were first transformed into tped format using plink v.107^48^ and then managed with R v.3.3.3^49^. For study cohort 2, the VCF data file was read into R using the seqminer library v.5.7^50^ and then managed similarly to study cohort 1. Positions that were identical over all of the included pairs were removed before analysis and plotting. The final pair comparison result matrix was plotted with the R library *lattice* v.0.20–35 function levelplot^51^. Recombination segments were identified based on visual inspection of pairwise comparison of genotype similarity plot.

### Statistical analysis

Study cohort 1 was used for analysing the association of GvHD clinical status with the extent of pairwise MHC mismatching. The analysis was performed by first counting the natural logarithm of total number of MHC mismatches in each HSCT sibling donor-recipient pair and then dividing the pairs into ‘high’ and ‘low’ mismatch groups. Due to the log transform, pairs with zero mismatches were omitted from analysis. Similarly, each pair was assigned into either a GvHD positive or negative group according to the recipient’s clinical GvHD gradus. The mismatch and GvHD categorized data were arranged into a contingency table to calculate the odds ratio (OR). The cutoff in defining the ‘high’ and ‘low’ mismatch pairs was varied from 0 to 6. The odds ratio was then calculated over the varying cutoff values and correlation between the cutoff and odds ratio was calculated with Kendall’s rank correlation. The correlation p-value was calculated with the R function *cor.test*. The linear relationship was visualized using regression line with its 95% confidence intervals on the odds ratio vs. cutoff plots. Furthermore, to estimate the robustness of the correlation at different definitions of GvHD positive and GvHD negative groups, either grade 0 subjects or grades 0–2 subjects were included in the negative acute GvHD group and either no GvHD or no/limited GvHD subjects were included in the negative chronic GvHD group. To estimate the possible effect of HLA-A,-B,-DRB1 mismatching that was known before the HSCT, the mismatched pairs were removed and the same analysis procedure was carried out for only the fully matched pairs. The analysis was done with R v.3.3.3^49^.

### Data availability statement

Data reported in this study is not available due to the limitations set by Ethical committee.

### Ethical approval and informed consent

All experiments were carried out in accordance with relevant guidelines and regulations defined in the Finnish legislation. The Ethical Committee of the Helsinki University Hospital and Valvira, the authority operating under the Ministry of Social Affairs and Health, have approved the study.

## Electronic supplementary material

Dataset 1

HLA allele mismatching in sibling donor HSCT pairs.

Recombination sites of the study cohorts 1 and 2.

Identity plot of HSCT pairs of study cohorts 1 and 2.

Demographics of the study cohort 1.
