# Supplementary material for: Hidden genomic MHC disparity between HLA-matched sibling pairs in hematopoietic stem cell transplantation
Source: Sci Rep. 2018 Mar 29;8:5396. doi: 10.1038/s41598-018-23682-y (PMC5876349; doi:10.1038/s41598-018-23682-y)
Supplement: Supplementary file 2 — HLA allele mismatching in sibling donor HSCT pairs. [file 41598_2018_23682_MOESM2_ESM.docx]

**Hidden genomic MHC disparity between HLA-matched sibling pairs in hematopoietic stem cell transplantation**

Satu Koskela^1^*, Jarmo Ritari^1^, Kati Hyvärinen^1^, Tony Kwan^2^, Riitta Niittyvuopio^3^, Maija Itälä-Remes^3^, Tomi Pastinen^2^, Jukka Partanen^1^

**Supplementary Table 2**. HLA alleles in 20 HSCT pairs of study cohorts 1 and 2 with mismatches in the seven classical HLA genes assigned by HLA imputation or sequence analysis.
