# Supplementary material for: Hidden genomic MHC disparity between HLA-matched sibling pairs in hematopoietic stem cell transplantation
Source: Sci Rep. 2018 Mar 29;8:5396. doi: 10.1038/s41598-018-23682-y (PMC5876349; doi:10.1038/s41598-018-23682-y)
Supplement: Supplementary file 3 — Recombination sites of the study cohorts 1 and 2. [file 41598_2018_23682_MOESM3_ESM.docx]

**Hidden genomic MHC disparity between HLA-matched sibling pairs in hematopoietic stem cell transplantation**

Satu Koskela^1^*, Jarmo Ritari^1^, Kati Hyvärinen^1^, Tony Kwan^2^, Riitta Niittyvuopio^3^, Maija Itälä-Remes^3^, Tomi Pastinen^2^, Jukka Partanen^1^

**Supplementary Table 3.** Recombination sites of the study cohorts 1 and 2.

| **ImmunoChip mismatches** | | | | | **ExomeSeq mismatches** | | | | |
| --- | --- | --- | --- | --- | --- | --- | --- | --- | --- |
|  |  |  |  |  |  |  |  |  |  |
| Sample | Recombination range | | Mismatches | | Sample | Recombination range | | Mismatches | |
|  | start | end | total | % of SNPs |  | start | end | total | % of SNPs |
| 2329 | 32022898 | 32996350 | 312 | 35.1 | 2468 | 29744809 | 30914751 | 1714 | 20.3 |
| 3205 | telomeric* | 30575603 | 816 | 32.4 | 5426 | 30930633 | 32075563 | 2683 | 30.0 |
|  | 32883450 | centromeric** | 259 | 49.3 |  | 32800224 | centromeric** | 913 | 23.5 |
| 4426 | 29199824 | 30987170 | 1040 | 37.1 | 5624 | 29747690 | 30992346 | 2327 | 26.0 |
|  | 32883450 | 33064605 | 108 | 40.3 |  | 32682394 | 33018439 | 525 | 15.6 |
| 4658 | telomeric* | 30048020 | 402 | 36.4 | 5728 | 29544825 | 30398186 | 1977 | 26.3 |
| 4754 | telomeric* | 29545208 | 139 | 42.8 | 5782 | 29744809 | 30938885 | 1253 | 14.6 |
|  | 30438226 | 31824828 | 525 | 36.1 |  | 32888566 | centromeric** | 1304 | 39.5 |
| 5366 | 30924024 | 32066819 | 558 | 52.5 | 6245 | telomeric* | 29537975 | 600 | 31.7 |
|  | 32883450 | centromeric** | 279 | 53.1 |  | 30077967 | 30734803 | 1055 | 31.8 |
| 1812 | 33008546 | centromeric** | 242 | 66.9 |  | 30951004 | 31976572 | 1958 | 22.7 |
| 3450 | 32883450 | 33064605 | 46 | 17.2 |  | 32021130 | 32203537 | 133 | 20.2 |
| 4152 | 32883450 | 33064605 | 46 | 17.2 |  | 33018439 | centromeric** | 492 | 20.5 |
| 5236 | 32744852 | centromeric** | 344 | 47.8 | 6385 | telomeric* | 30041509 | 2494 | 34.7 |
| 2934 | 32883450 | centromeric** | 216 | 41.1 | 6768 | telomeric* | 29548775 | 628 | 32.4 |
| 3446 | 32900718 | 33008546 | 46 | 30.3 |  | 30120912 | 32212233 | 3330 | 24.5 |
| 4706 | 32967741 | centromeric** | 71 | 16.2 |  | 32743298 | 33018377 | 635 | 29.4 |
| 3803 | telomeric* | 29489048 | 144 | 53.5 | 5672 | 33182870 | centromeric** | 92 | 15.9 |
| 4205 | 29582212 | 30792364 | 573 | 24.5 | 6907 | 33079766 | centromeric** | 359 | 25.5 |
|  | 31392906 | 32057627 | 173 | 42.3 | 5559 | 32762792 | 33017486 | 273 | 14.6 |
|  |  |  |  |  |  | 33242825 | centromeric** | 98 | 21.8 |
|  |  |  |  |  | 7086 | 29033053 | 29549261 | 357 | 19.8 |
| * the telomeric boundary set at 33.5 Mbp | | | |  |  |  |  |  |  |
| ** the centromeric boundary set at 29.0 Mbp | | | | |  |  |  |  |  |
