# Supplementary material for: Hidden genomic MHC disparity between HLA-matched sibling pairs in hematopoietic stem cell transplantation
Source: Sci Rep. 2018 Mar 29;8:5396. doi: 10.1038/s41598-018-23682-y (PMC5876349; doi:10.1038/s41598-018-23682-y)
Supplement: Supplementary file 4 — Identity plot of HSCT pairs of study cohorts 1 and 2. [file 41598_2018_23682_MOESM4_ESM.docx]

**Hidden genomic MHC disparity between HLA-matched sibling pairs in hematopoietic stem cell transplantation**

Satu Koskela^1^*, Jarmo Ritari^1^, Kati Hyvärinen^1^, Tony Kwan^2^, Riitta Niittyvuopio^3^, Maija Itälä-Remes^3^, Tomi Pastinen^2^, Jukka Partanen^1^

**Supplementary Figure 1. Identity plot of HSCT pairs of study cohorts 1 and 2**. The HSCT pairs are shown in numerical order on the X-axis. Green colour depicts matched SNPs, yellow depicts mismatched SNPs and red colour indicate missing results or poor quality SNPs. **A)** Identical-by-state status of 5137 SNPs covering the entire MHC gene complex by an Immunochip array in 261 HSCT sibling pairs (study cohort 1). The Y-axis shows a segment of chromosome 6p21 encompassing positions 29,002,062 bp (telomeric to HLA-F) to 33,496,714 bp (centromeric to DPB1). **B)** Identical-by-state status of 89 sibling HSCT pairs sequenced by ImmunoSeq platform covering the 4 Mb MHC region (study cohort 2). The Y-axis shows a segment of chromosome 6p21 encompassing positions 29,000,825 bp (telomeric to HLA-F) to 33,477,140 bp (centromeric to DPB1).

**A)**


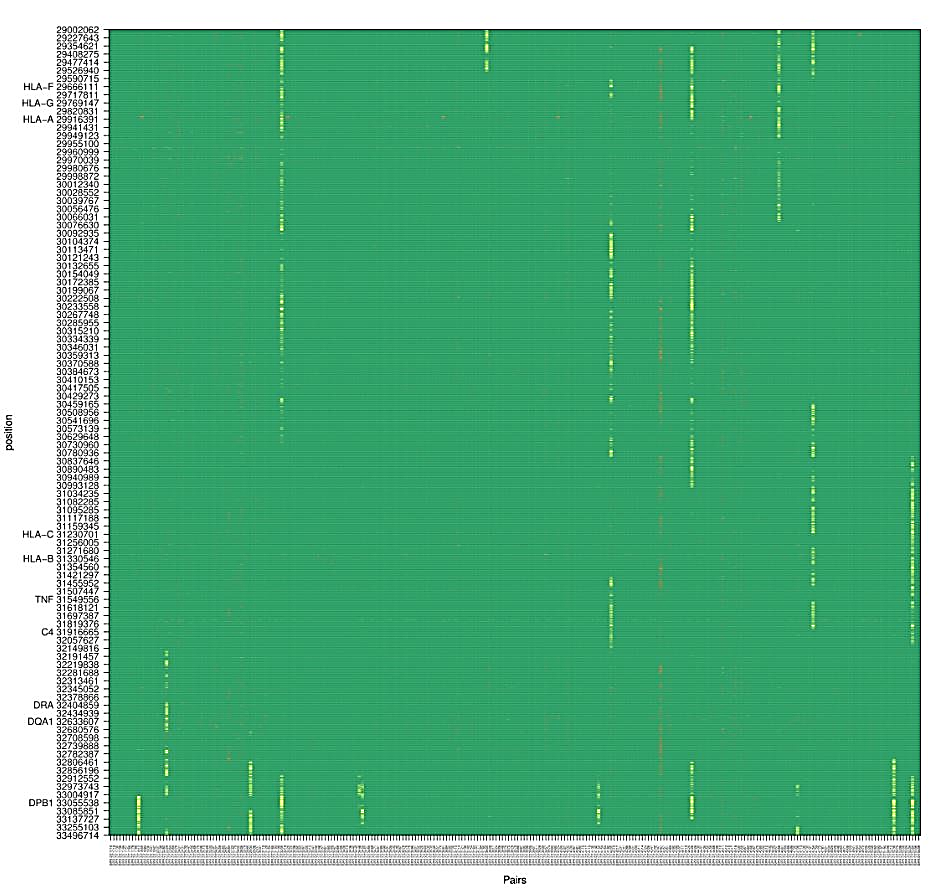


**
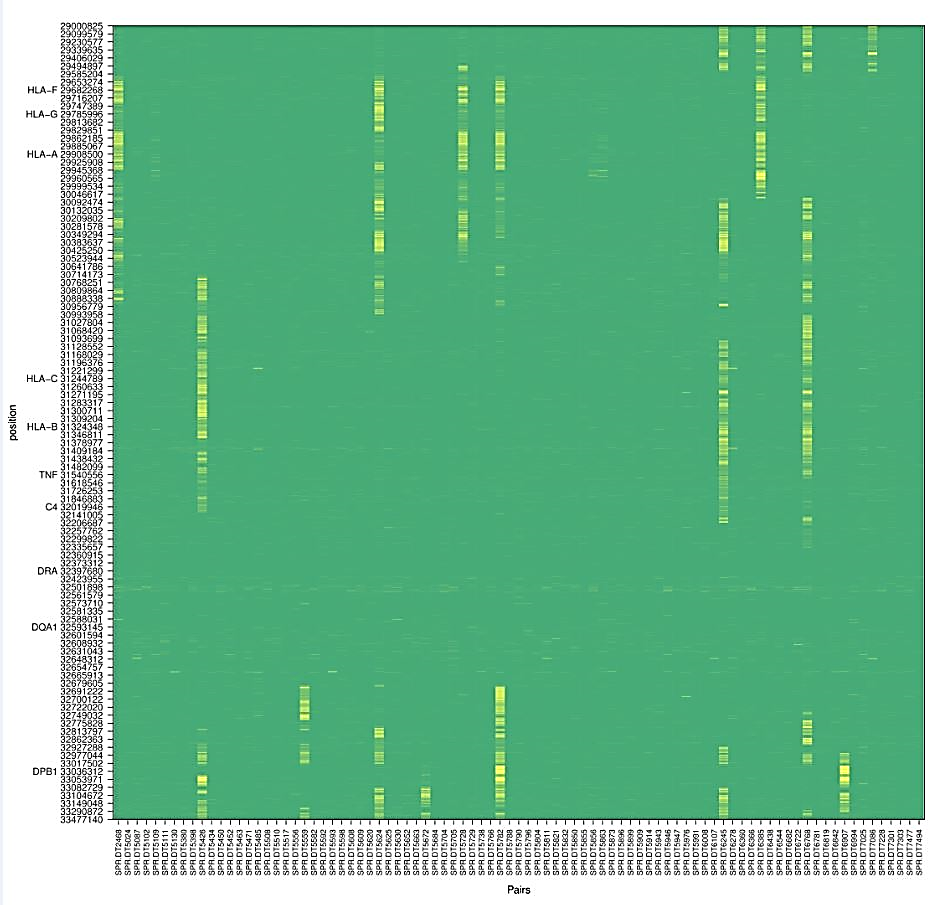
B)**
