# Supplementary material for: Hidden genomic MHC disparity between HLA-matched sibling pairs in hematopoietic stem cell transplantation
Source: Sci Rep. 2018 Mar 29;8:5396. doi: 10.1038/s41598-018-23682-y (PMC5876349; doi:10.1038/s41598-018-23682-y)
Supplement: Supplementary file 5 — Demographics of the study cohort 1. [file 41598_2018_23682_MOESM5_ESM.docx]

**Hidden genomic MHC disparity between HLA-matched sibling pairs in hematopoietic stem cell transplantation**

Satu Koskela^1^*, Jarmo Ritari^1^, Kati Hyvärinen^1^, Tony Kwan^2^, Riitta Niittyvuopio^3^, Maija Itälä-Remes^3^, Tomi Pastinen^2^, Jukka Partanen^1^

| **Supplementary Table 4. Demographics of the study cohort 1** | | | |
| --- | --- | --- | --- |
|  |  |  |  |
| **Transplants (n=261)** | | | |
| **Age** |  | **y, median** | **y, range** |
|  | recipient | 49.0 | 18–65 |
|  | donor | 46.5 | 4–65 |
| **Year of transplantation** | | **n** | **%** |
|  | 1993-1997 | 30 | 11.5 |
|  | 1998-2000 | 52 | 20.0 |
|  | 2001-2003 | 120 | 46.0 |
|  | 2004-2006 | 55 | 21.1 |
|  | missing | 4 | 1.5 |
| **Patient-donor gender** | | **n** | **%** |
|  | male-male | 71 | 27.2 |
|  | male-female | 57 | 21.8 |
|  | female-female | 59 | 22.6 |
|  | female-male | 70 | 26.8 |
|  | missing | 4 | 1.5 |
| **Diagnosis** | | **n** | **%** |
|  | acute myeloid leukemia | 75 | 28.7 |
|  | acute lymphoblastic leukemia | 39 | 14.9 |
|  | chronic myeloid leukemia | 36 | 13.8 |
|  | myelodysplastic syndrome | 21 | 8.0 |
|  | Hodgkin’s lymphoma | 0 | 0.0 |
|  | non-Hodgkin’s lymphoma | 12 | 4.6 |
|  | myeloma | 50 | 19.2 |
|  | aplastic anemia | 5 | 1.9 |
|  | other malignancies | 19 | 7.3 |
|  | missing | 4 | 1.5 |
| **Stem cell source** | | **n** | **%** |
|  | bone marrow | 134 | 51.3 |
|  | peripheral blood | 123 | 47.1 |
|  | missing | 4 | 1.5 |
| **Conditioning regimen** | | **n** | **%** |
|  | myeloablative | 197 | 75.5 |
|  | reduced intensity conditioning | 60 | 60 |
|  | missing | 4 | 1.5 |
| **aGvHD grading** | | **n** | **%** |
|  | grade 1 | 38 | 14.6 |
|  | grade 2 | 20 | 7.7 |
|  | grade 3 | 19 | 7.3 |
|  | grade 4 | 6 | 2.3 |
|  | no aGvHD | 172 | 65.9 |
|  | missing | 6 | 2.3 |
| **cGvHD grading** | | **n** | **%** |
|  | limited | 56 | 21.5 |
|  | extensive | 67 | 25.7 |
|  | no cGvHD | 114 | 43.7 |
|  | missing | 24 | 9.2 |
